# Knowledge-based biomedical word sense disambiguation: comparison of approaches

**DOI:** 10.1186/1471-2105-11-569

**Published:** 2010-11-22

**Authors:** Antonio J Jimeno-Yepes, Alan R Aronson

**Affiliations:** 1 National Library of Medicine, 8600 Rockville Pike, Bethesda, MD 20894, USA

## Abstract

**Background:**

Word sense disambiguation (WSD) algorithms attempt to select the proper sense of ambiguous terms in text. Resources like the UMLS provide a reference thesaurus to be used to annotate the biomedical literature. Statistical learning approaches have produced good results, but the size of the UMLS makes the production of training data infeasible to cover all the domain.

**Methods:**

We present research on existing WSD approaches based on knowledge bases, which complement the studies performed on statistical learning. We compare four approaches which rely on the UMLS Metathesaurus as the source of knowledge. The first approach compares the overlap of the context of the ambiguous word to the candidate senses based on a representation built out of the definitions, synonyms and related terms. The second approach collects training data for each of the candidate senses to perform WSD based on queries built using monosemous synonyms and related terms. These queries are used to retrieve MEDLINE citations. Then, a machine learning approach is trained on this corpus. The third approach is a graph-based method which exploits the structure of the Metathesaurus network of relations to perform unsupervised WSD. This approach ranks nodes in the graph according to their relative structural importance. The last approach uses the semantic types assigned to the concepts in the Metathesaurus to perform WSD. The context of the ambiguous word and semantic types of the candidate concepts are mapped to Journal Descriptors. These mappings are compared to decide among the candidate concepts. Results are provided estimating accuracy of the different methods on the WSD test collection available from the NLM.

**Conclusions:**

We have found that the last approach achieves better results compared to the other methods. The graph-based approach, using the structure of the Metathesaurus network to estimate the relevance of the Metathesaurus concepts, does not perform well compared to the first two methods. In addition, the combination of methods improves the performance over the individual approaches. On the other hand, the performance is still below statistical learning trained on manually produced data and below the maximum frequency sense baseline. Finally, we propose several directions to improve the existing methods and to improve the Metathesaurus to be more effective in WSD.

## Background

### Introduction

Word sense disambiguation (WSD) algorithms attempt to select the proper sense of ambiguous terms in text. A word is ambiguous when it has more than one sense, which is determined based on the context in which the word is used. WSD is an intermediary step within information retrieval and information extraction. Thus, improvement in WSD will help, for instance, to produce better annotation tools like MetaMap [1], improve automatic indexing [2] and other text mining tasks.

In the following examples, the word *culture *has two senses: in the first sentence it stands for *laboratory culture *while the seconds stands for *anthropological culture*. The examples are sentences from MEDLINE^® ^citations, which is the largest bibliographic database in the biomedical domain with citations from around 5,000 journals, with their PUBMED^® ^identifiers (PMIDs).

1. PMID: 9422921-Of the intraocular samples taken from post-surgical and post-traumatic cases, 10/27 (37%) and 3/5 (60%) were **culture **positive, respectively.

2. PMID: 9467727-Through mutual interaction biology in humans becomes **culture**, and vice versa, culture opens and stimulates the neural passages of the brains, accounting thus for the varieties of brains in humans, and for cultural diversity.

The UMLS^® ^(Unified Medical Language System) Metathesaurus^® ^[3,4] is the largest biomedical thesaurus available, consisting of over 8 million medical terms collected from more than 100 resources. Several efforts exist to map the UMLS^® ^to text, (e.g. MetaMap [1] and Whatizit [5]). The UMLS 2009AB version has at least 24.000 ambiguous terms, i.e. where a given term is part of more than one concept unique identifier (CUI) in the Metathesaurus. These ambiguous cases increase if we consider term variability introduced by matching algorithms. All UMLS concepts are assigned one or more broader categories called semantic types. This version of the Metathesaurus is available from [6]. We have used the standard installation available in the 2009AB distribution.

For each term in the Metathesaurus, we have calculated the number of distinct concepts to which they are assigned. If the number of distinct concepts assigned to a term is bigger than one, then we consider it to be ambiguous. Each one of these senses is assigned a different concept identifier in the Metathesaurus.

Table 1 shows the distribution of types with the most cases of ambiguity. Semantic types related to proteins, genes and clinical contain the largest number of ambiguous cases.

**Table 1:** Top 10 most ambiguous semantic types

Frequency	Type	Description
8,688	T028	Gene or Genome
4,089	T116	Amino Acid, Peptide, or Protein
3,534	T201	Clinical Attribute
2,189	T200	Clinical Drug
1,969	T047	Disease or Syndrome
1,691	T123	Biologically Active Substance
1,408	T170	Intellectual Product
1,278	T121	Pharmacologic Substance
1,252	T126	Enzyme
1,218	T129	Immunologic Factor

Table 2 presents the top terms in MEDLINE from the ambiguous terms in the Metathesaurus. Compared to the semantic type statistics, it seems that semantic types other than proteins or genes dominate the most interesting cases. We observe that terms like *study *can be mapped to 6 concepts, showing the complexity of the UMLS content.

**Table 2:** Top 10 most ambiguous terms in MEDLINE

Frequency	Term	Amb. level
3,215,158	study	6
2,122,371	treatment	4
2,064,598	all	6
1,955,592	2	5
1,945,251	1	5
1,872,536	other	44
1,795,137	had	2
1,762,387	effect	2
1,757,672	can	11
1,755,725	cell	4

In addition, the UMLS requires preprocessing since some terms provided by the constituent vocabularies provide some terms like general English terms and numbers which might be difficult to deal with and which might not be of interest in the biomedical context (*all*, *other*, *had*, *can*, ...). The term *other *is the term with the largest ambiguity level. The Metathesaurus concepts linked to this term denote the use of the term in specific contexts (e.g. *Other-Diagnosis Classification*). Numbers are also ambiguous in the UMLS and denote either the position number or a position in a set of entities (e.g. *Grade 1 *or *Tooth 1 *). Even though the ambiguity levels for numbers *1 *and *2 *are the same, the concepts they are mapped to are completely different. Several procedures [7-9] have already been studied to perform a cleanup of these cases.

Usually, WSD techniques developed using statistical learning achieve better performance compared to knowledge-based approaches [10]. On the other hand, building a manually annotated corpus, as required by statistical learning, to cover all the concepts in the UMLS Metathesaurus is expensive and infeasible. State of the art knowledge-based approaches rely on graph theory [11] which have interesting performance but are still far from the maximum frequency sense baseline or the statistical learning approaches.

### Related work

Previous work in WSD for the UMLS includes knowledge-based and supervised methods. Among the knowledge-based methods we find the Journal Descriptor Indexing method [12] and several based on graph algorithms [13]. Machine learning algorithms have been explored in several studies where alternative combinations of features are compared [14-16]; these studies obtain a performance of over 0.86 in terms of accuracy using the collection prepared by Weeber et al. [17].

Related work in the biomedical domain shows that statistical learning performs better than unsupervised or knowledge-based ones. Existing corpora in the biomedical domain [13,17] cover just a small number of terms and senses compared to the content of the UMLS Metathesaurus. Manually extending existing corpora to cover the entire UMLS Metathesaurus does not seem to be feasible.

The idea of generating corpora automatically to perform WSD has already been presented in the WSD literature. Leacock et al. [18] used monosemous relatives and co-occurrences to retrieve training data. Their automatically generated dataset showed promising results but not as good as training with manually generated data. Agirre and Martinez [19] built corpora for WSD based on the Web. In their work, evaluated on Senseval-2 [20], they show the feasibility of building such a corpus and pointed out that better results are obtained on a corpus in which the number of examples per sense is biased following the sense distribution in the manually annotated set. Unfortunately, the sense distribution is difficult to obtain automatically.

The automatic acquisition of corpora to perform WSD has already been successfully used in the biomedical domain to disambiguate acronyms [21]. In this case, the occurrences of long forms and acronyms are located using pattern matching. The examples are collected and processed to perform learning based on the SVM learning algorithm.

In this paper, we compare several knowledge-based methods which do not require training on manually annotated data.

## Methods

We compare four knowledge-based methods which have different assumptions on how the terms should be disambiguated. These methods use the UMLS Metathesaurus as the knowledge source to perform disambiguation. In our work a word sense is equivalent to a concept in the Metathesaurus. Each concept is uniquely identified by a Concept Unique Identifier (CUI).

The first approach compares the overlap of the context where the ambiguous word appears to the candidate concepts based on a representation built out of the definition, synonyms and related terms.

The second approach exploits the structure of the Metathesaurus network of relations on a graph-based method to perform unsupervised WSD. This approach ranks nodes in the graph according to their relative structural importance.

The third method collects training data using PUBMED queries built out of the monosemous synonyms and related terms for each one of the senses of the ambiguous term. A Naïve Bayes classifier is trained on the retrieved citations and used to disambiguate the ambiguous word instances.

The last approach uses the semantic types assigned to the concepts in the Metathesaurus to perform WSD. The context of the ambiguous word and semantic types of the candidate concepts are mapped to Journal Descriptors. The Journal Descriptors are compared to decide among the candidate concepts.

All the approaches compared in this paper rely on the UMLS as the knowledge source to perform disambiguation. In the following section, the UMLS is introduced. Then, the different approaches are presented, including two approaches to combine the approaches, and finally the data set used for evaluation is described.

### UMLS

The NLM's UMLS provides a large resource of knowledge and tools to create, process, retrieve, integrate and/or aggregate biomedical and health data. The UMLS has three main components:

• Metathesaurus, a compendium of biomedical and health content terminological resources under a common representation which contains lexical items for each one of the concepts and relations among them. In the 2009 AB version it contains over a million concepts.

• Semantic network, which provides a categorization of Metathesaurus concepts into semantic types. In addition, it includes relations among semantic types.

• SPECIALIST lexicon, containing lexical information required for natural language processing which covers commonly occurring English words and biomedical vocabulary.

Concepts in the Metathesaurus denote possible senses that a term may have in the Metathesaurus. Concepts are assigned a unique identifier (CUI) which has linked to it a set of terms which denote alternative ways to represent the concept, for instance, in text. These terms, depending on the availability, are represented in several languages. Only English terms are used in this work. Concepts are assigned one or more semantic types. Concepts may have a definition linked to them and sometimes more than one from multiple sources. Relations between concepts are often available. All the information about a concept can be traced back to the resource from where it was collected.

The concept with CUI *C0009264 *denotes the idea of *cold temperature*. According to the Metathesaurus, terms like *cold*, *cold temperature *and *low temperature *could be used to express this idea. In addition, two definitions are available for this concept (from MeSH and from the NCI Thesaurus), e.g. *An absence of warmth or heat or a temperature notably below an accustomed norm*. Several relations are available, linking the concept to sibling concepts (*heat*), hypernyms (*temperature*) and non-taxonomically related (*cold storage*, *cryotherapy*).

### Machine Readable Dictionary approach

In our first WSD approach, the context words surrounding the ambiguous word are compared to a profile built from each of the UMLS concepts linked to the ambiguous term begin disambiguated. This approach has been previously used by McInnes [22] in the biomedical domain with the NLM WSD corpus.

This algorithm can be seen as a relaxation of Lesk's algorithm [23], which is very expensive since the sense combination might be exponentially large even for a single sentence. Vasilescu et al. [24] have shown that similar or even better performance might be obtained disambiguating each ambiguous word separately.

A concept profile vector has as dimensions the tokens obtained from the concept definition or definitions if available, synonyms, and related concepts excluding siblings.

Stop words are discarded, and Porter stemming is used to normalize the tokens. In addition, the token frequency is normalized based on the inverted *concept *frequency so that terms which are repeated many times within the UMLS will have less relevance.

A context vector for an ambiguous term includes the term frequency; stop words are removed and the Porter stemmer is applied. The word order is lost in the conversion.

In this machine readable dictionary approach (MRD), vectors of concept profiles *c *linked to an ambiguous word *w *in set *C_w _*and word contexts *cx *are compared using cosine similarity as shown in equation 1; the concept with the highest cosine similarity is selected.

(1) $$MRD(c)=\;\underset{c\in C_{w}}{argmax}\frac{c\cdot cx}{\left|c\right|\left|cx\right|}$$

### Page Rank WSD implementation

This second approach combines the context of the word with the chances of selecting the concept based on the topology of the network of the resource used for disambiguation. The algorithm was developed by Agirre and Soroa [11]. It is inspired by the Google Page Rank algorithm, which is used to encode word sense dependencies using random walks on graphs.

In this approach the knowledge resource is represented as follows. Let *G *be a graph with *N *vertices *v*_1 _, ..., *v_N _*, *d_i _*be the outdegree of node *i*; let matrix *M *be an *N *× *N *transition probability matrix. $M_{ij}=\frac{1}{d_{i}}$. To estimate the PageRank vector *Pr *over *G *requires solving equation 2, where *v *is an *N *× 1 vector of elements $\frac{1}{N}$ and *c *is a *damping factor*.

(2) Pr=cMPr+(1−c)v

The 1999 Metathesaurus has been processed as follows to prepare it for the PageRank algorithm. A dictionary file (containing terms and pointers to concepts) and a relation file are produced according to Agirre and Soroa's implementation.

**Figure 1:**
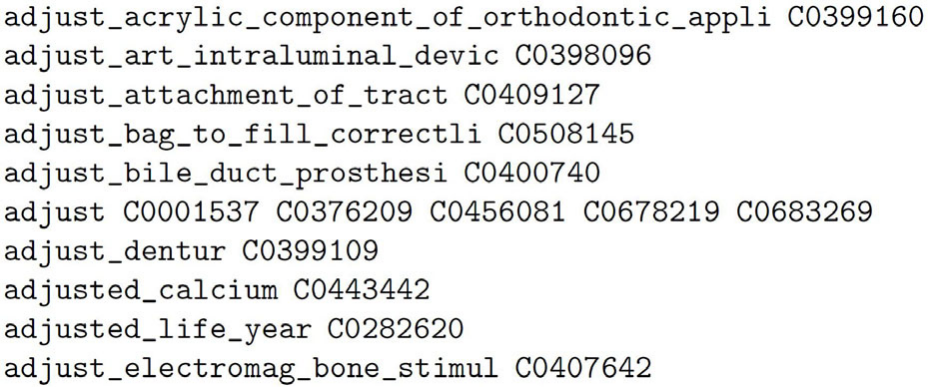
**Example of dictionary file used in the Page Rank method**. The first field is a word in the dictionary. The remaining fields are the possible identifiers in the Metathesaurus. If more than one identifier appears, it means that the word is ambiguous and the identifiers are the possible candidates.

To generate the dictionary file, the terms in the *str *field of the MRCONSO table [25] in the Metathesaurus are normalized using the Porter stemmer. Spaces in multi-word terms are replaced by underscore characters. Words with multiple concept identifiers are grouped, and the collected concept identifiers are shown one after the other. Words with less than 3 characters or more than 50 characters are not considered. In the 1999 Metathesaurus, ambiguous terms have, in addition, entries assigning a sequential number (e.g. *adjustment_ < 1 >, adjustment*_* < 2 >*, ...). These entries are ignored since there is always an entry with the ambiguous word without the sequence number. Terms with parentheses or square brackets are ignored, too. Part-of-speech annotation of words is not available in the Metathesarus as in WordNet, so this information is not used. A sample of the dictionary file can be found in figure 1.

**Figure 2:**
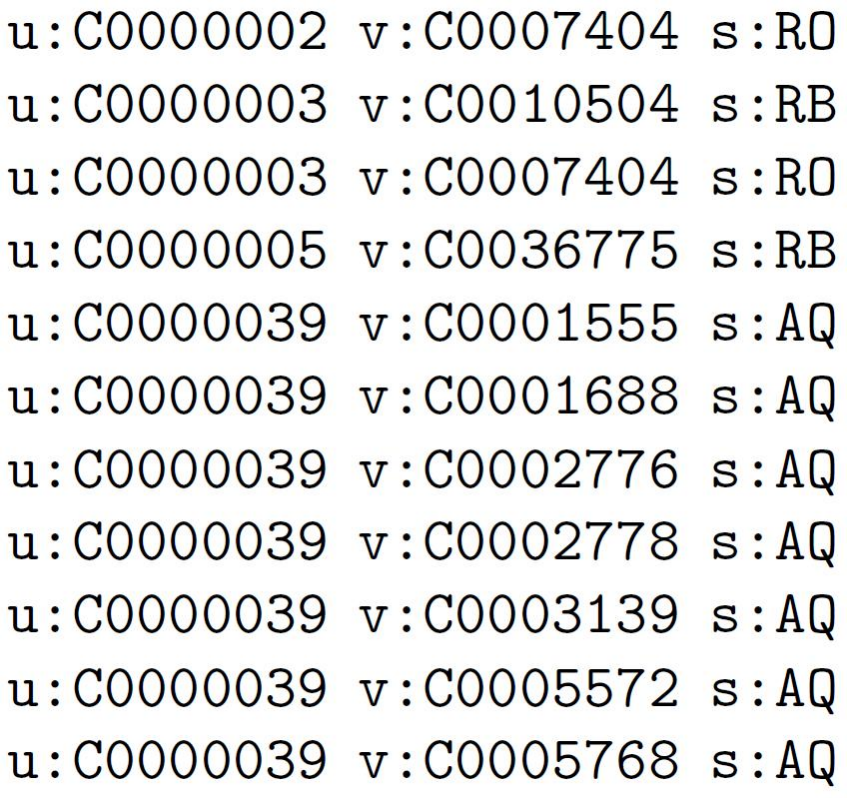
**Example of relation file used in the Page Rank method**. The u and v fields indicate the origin and destination of the link respectively. The s field indicates the relation as it appears in the MRREL file [26] (AQ-allowed qualifier, RB - has a broader relationship, RO - has relationship other than synonyms, narrower or broader).

The relation file is prepared as follows. Relations are available from the MRREL table [26] in the Metathesaurus. In this table, relations between Metathesaurus concepts include the two concepts (*cui1*, *cui2 *fields) involved and the type of relation between the entities (*rel *field). The information concerning these relations are extracted and converted to an appropriate format. A sample of the relation file can be in figure 2.

**Figure 3:**
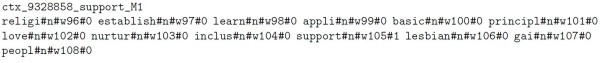
**Example of instance to be disambiguated by the Page Rank method**. The first line is used to identify the citation (PMID), the ambiguous word and the correct sense of the ambiguous word. In the second line, which shows only part of the citation, each word is composed of: stemmed term, a part-of-speech (to be ignored in our experiments), the word count and a flag which indicates if the word should be disambiguated (0 - do not disambiguate, 1 - disambiguate).

The instances to be disambiguated follow a process similar to that for Metathesaurus terms. Tokens in the context of the ambiguous words and in the dictionary file might not match in some cases since Metathesaurus terms might contain multiple words. To map the Metathesaurus multi-term words to text we have used MetaMap [1]. MetaMap is available as a batch mode tool [27]. This service handles several Metathesaurus versions including the 1999 one. Once the multi-word terms are grouped, stopwords are removed and Porter stemming is applied. An example can be found in figure 3.

### Automatic corpus extraction from MEDLINE

In this third approach, corpora to train for statistical learning algorithms for ambiguous terms are prepared by retrieving documents from a large corpus. For our large corpus, we use MEDLINE [28]. The Metathesaurus is used to obtain information related to the candidate concepts linked to an ambiguous term.

Queries are generated using English *monosemous relatives *[18] of the candidate concepts which, potentially, have an unambiguous use in MEDLINE. The list of candidate relatives include synonyms and terms from related concepts as shown in the UMLS section above. In our work with the Metathesaurus, we consider a term as monosemous if it is only assigned to one concept. This means that *cold *is ambiguous since it is linked to more than one concept in the Metathesaurus while the term *cold storage *is monosemous because it is only linked to concept with CUI *C0010405*.

Further filtering is applied to the selected monosemous terms. Long terms (more than 50 characters) are not considered since these are unlikely to appear in MEDLINE. This avoids having unnecessarily long queries which could be problematic with retrieval systems. Very short terms (less than 3 characters) and numbers are not considered to avoid almost certain ambiguity. A standard stop word list is used to remove uninformative English terms.

**Figure 4:**
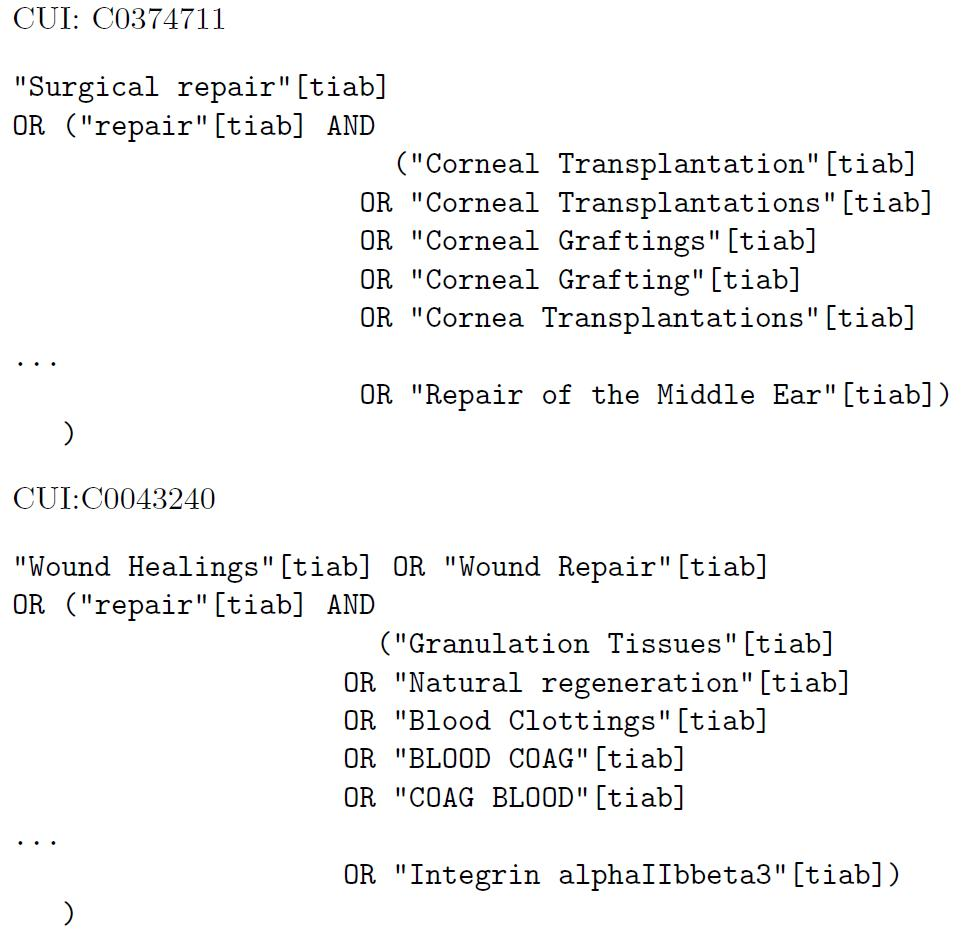
**Query example for term repair using synonyms and related concepts**.

We have used EUtils [29] from PUBMED [30] as the search engine to retrieve documents from MEDLINE. The query language used by PUBMED is based on Boolean operators and allows for field search, e.g. it allows searching a specific term within the metadata. Monosemous synonyms are added to the query and joined with the OR operator. Monosemous terms from related concepts are combined with the AND operator with the ambiguous term assuming one sense per collocation, then combined with monosemous synonyms using the OR operator. In order to retrieve documents where the text (title or abstract of the citation) contains the query terms, the *[tiab] *search field is used. Quotes are used to find exact mentions of the terms and increase precision. Examples of queries for the ambiguous term *repair*, with concept identifiers *C0374711 *and *C0043240*, using monosemous relatives are found in figure 4.

Documents retrieved using PUBMED are assigned to the concept which was used to generate the query. If no documents are returned for a given query, the quotes are replaced by parentheses to allow finding the terms in any position in the title or abstract text. The retrieved documents are used to create training examples for each sense.

This corpus is used to train a statistical learning algorithm, e.g. Naïve Bayes. Disambiguation is performed using the trained model with new disambiguation examples. In the results presented in this work, the trained model is evaluated against a manually annotated set from which accuracy values are recorded. We have evaluated several limits on the number of retrieved documents. Since there is not a significant difference in performance, 100 documents are collected from MEDLINE for each concept identifier.

### Journal Descriptor Indexing

In domain-specific contexts, senses of terms might be assigned to different domains or categories. In the Metathesaurus, concepts are assigned one or more semantic categories from the semantic types. For instance, in the Metathesarus the term *cold *could be classified as *natural phenomenon or process*, a *disease or syndrome *or a *therapeutic procedure*.

Disambiguation using semantic categories is done assigning these categories to the context where the ambiguous string appears, possibly with a score representing our confidence, and then assigning the sense matching the assigned semantic category. The Journal Descriptor Indexing (JDI) method developed by Humphrey et al. [12] is an example of this technique in the biomedical domain. The JDI disambiguation method is the disambiguation method available from MetaMap. In addition, it is available as part of the SPECIALIST Text Categorization tools [31].

This technique uses the semantic types assigned to Metathesaurus concepts to perform disambiguation. Journal Descriptors (JD) are general MeSH headings assigned to the journals in MEDLINE.

The JDI technique will assign a score to the semantic types which will allow selecting the highest ranking semantic type of the target ambiguous word. The selected semantic type is used to identify the proper concept in the Metathesaurus under the assumption that each ambiguous word is assigned to a distinct semantic type. The score is estimated by comparing the JD indexing of the context of the ambiguous word and the pre-calculated JD indexing of the semantic types using cosine similarity. JD indexing is represented as a vector of JDs with a score. This score represents the confidence in the indexing of the JD. JD indexing relies on building for each JD a vector of words based on a training data of citations extracted from MEDLINE. As mentioned above, journals in MEDLINE are indexed using JDs. The citations in those journals that are in MEDLINE are used to build a word vector for each JD. Words in the vector are assigned probabilities estimated by counting the number of times a word is related to a JD divided by the total number of citations.

Pre-calculated JD indexing of the semantic types is built for each semantic type comparing a word vector of semantic types to the JD word vectors. Words are extracted from the concepts in the Metathesaurus assigned to each semantic type. Detailed examples of use are available in [12].

Approaches working on semantic categories rely on a distinct assigment of these categories to the senses of the ambiguous word. These approaches cannot disambiguate cases where the concepts linked to the ambiguous word are assigned the same semantic type. For instance, in the Metathesaurus the term *cold *is linked to two concepts within the *Disease or Syndrome *semantic types: *common cold, C0009443 *and *chronic obstructive airway disease, C0024117*.

### Combination of approaches

We have shown several different knowledge-based approaches with different views on the disambiguation problem. These approaches can be combined since they might complement each other. The combination of the approaches has been done either by maximum vote (Combine vote) or by linear combination of the prediction probability or score assigned to the senses (Comb. linear).

The *Combine vote *method for a given disambiguation example collects the concept predicted by each approach. Then for each concept the number of votes are counted and the concept with the maximum number of votes is selected. Ties are solved by random selection of one of the concepts.

The *Comb. linear *method for a given disambiguation example collects for each candidate concept the score provided by each approach and returns the linear combination of these scores. Then, the concept with the highest score is selected. The score provided by each method score denotes the confidence of the method in predicting the given concept for the disambiguation example. All the knowledge-based WSD approaches considered in this work provide a numerical value between 0 and 1. The contribution of each method might be weighted according to the confidence of each approach.

### Evaluation set

The NLM WSD data set [17,32] has been used to conduct the experiments. This set contains 50 ambiguous terms which have been annotated with a sense number. Each sense number has been related to UMLS semantic types. 100 manually disambiguated cases are provided for each term. In case no UMLS concept is appropriate, *None of the above *has been assigned in the NLM WSD.

The selection of the 50 ambiguous words was based on an ambiguity study of 409,337 citations added to the database in 1998. MetaMap was used to annotate UMLS concepts in the titles and abstracts based on the 1999 version of the UMLS. 50 highly frequent ambiguous strings were selected for inclusion in the test collection. Out of 4,051,445 ambiguous cases found in these citations, 552,153 cases are represented by these 50 terms. This means that a large number of ambiguous cases can be solved dealing with these highly frequent cases. A team of 11 people annotated the ambiguous cases with Metathesaurus entries. The data set is available from [33].

No CUIs were provided with the set, but there is a mapping to UMLS CUIs for the 1999 version of the UMLS Metathesaurus. In addition, from the same site [32] it is possible to obtain the version of the UMLS used for the development of the NLM WSD data set which we have used in our work. Recently, a mapping to the 2007AB version of the Metathesaurus has been made available. This data set might be used to test the difference in performance of the same WSD method with different version of the Metathesaurus.

We have considered the same setup as Humphrey et al. [12] and discarded the *None of the above *category. Since the ambiguous term *association *has been assigned entirely to *None of the above*, it has been discarded. This means that we will present results for 49 out of the 50 ambiguous terms.

## Results and Discussion

Results using the NLM WSD data set are presented in terms of accuracy, defined in equation 3, where an instance is an example of an ambiguous word to disambiguate.

We have used several baselines which allow comparing different assumptions. One baseline is maximum frequency sense (MFS), which is standard in WSD evaluation. The other baseline is based on Naïve Bayes; 10-fold cross-validation sampling is used.

Words occurring in the text of the citation, where the ambiguous word appears, are used as the context of the ambiguous word. All the algorithms have used the text in the citation (title and abstract) as context to perform disambiguation.

The JDI approach, as mentioned above, cannot deal with cases where candidate concepts have the same semantic type. Due to this, a total of 4 ambiguous words are discarded from our evaluation set when using this method: *cold*, *man*, *sex *and *weight*. In tables 3 and 4, averages over the whole set (*Accuracy All*) and the JDI set (*Accuracy JDI set*) are presented. Table 3 shows the results comparing the use of the automatic extracted corpus (AEC), machine readable dictionary (MRD), Page Rank (PPR), JDI and the baselines. Table 4 shows a per-word result which allows a finer-grained evaluation.

**Table 3:** NLM WSD results: method comparison

1999	Accuracy all	Accuracy JDI set
MRD	0.6389	0.6526
PPR s	0.5826	0.5867
AEC	0.6836	0.6932
JDI		0.7475

CombSW	0.7626	0.7794
CombV	0.7601	0.7739

MFS	0.8550	0.8669
NB	**0.8830**	**0.9063**

MRD stands for Machine Readable Dictionary, PPR s stands for Page Rank and MetaMap with Strict model, AEC stands for Automatic Extracted Corpus, JDI stands for Journal Descriptor Indexing, CombSW stands for weighted linear combination, CombV stands for voting combination, MFS stands for Maximum Frequency Sense and NB stands for Naïve Bayes.

**Table 4:** Accuracy results per word

Word	F Max	Total	MFS	NB	AEC	MRD	PPR s	JDI	CombSW	CombV
adjustment	62	93	0.6667	0.7634	0.6237	0.2308	0.3226	0.6923	0.6882	0.5269
blood pressure	54	100	0.5400	0.5700	0.3700	0.4343	0.4600	0.2020	0.3838	0.4444
cold	86	95	0.9053	0.9263	0.3895	0.6044	0.9158		0.3895	0.7895
Condition	90	92	0.9783	0.9783	0.7065	0.3370	0.9121	0.8370	0.7802	0.6923
culture	89	100	0.8900	0.9300	0.6000	0.8200	0.1212	0.9700	1.0000	0.5455
degree	63	65	0.9692	0.9692	0.8923	0.4923	0.9692	0.7077	0.8769	0.8154
depression	85	85	1.0000	1.0000	0.9529	0.9941	0.9294	0.9176	0.9647	0.9882
determination	79	79	1.0000	1.0000	0.1392	0.9936	0.0000	1.0000	0.9620	0.1392
discharge	74	78	0.9487	0.9867	0.7067	0.9861	0.8800	0.5556	0.7067	0.9600
energy	99	100	0.9900	0.9900	0.4000	0.4536	0.1000	0.7732	0.4600	0.5400
evaluation	50	100	0.5000	0.7800	0.5000	0.5800	0.5000	0.5800	0.5200	0.5000
extraction	82	87	0.9425	0.9425	0.7471	0.2907	0.5747	0.9535	0.9770	0.8621
failure	25	29	0.8621	0.8621	0.8621	0.5862	0.8621	1.0000	0.8621	1.0000
fat	71	73	0.9726	0.9726	0.8356	0.9718	0.2029	0.9296	0.9130	0.8406
fit	18	18	1.0000	0.8330	0.8889	0.8387	1.0000	1.0000	0.8889	1.0000
fluid	100	100	1.0000	0.9710	0.4800	0.6082	0.5354	0.3608	0.4848	0.3535
frequency	94	94	1.0000	0.9690	0.6064	0.9362	1.0000	0.1809	0.6277	0.8085
ganglion	93	100	0.9300	0.9500	0.8600	0.9565	0.3838	0.9130	0.8788	0.8586
glucose	91	100	0.9100	0.9100	0.7800	0.2755	0.9200	0.7347	0.7800	0.3900
growth	63	100	0.6300	0.7300	0.3700	0.6700	0.3700	0.6500	0.5500	0.6600
immunosuppression	59	100	0.5900	0.7900	0.5700	0.4896	0.4646	0.7083	0.5960	0.6465
implantation	81	98	0.8265	0.9796	0.9490	0.8316	0.8367	0.9053	0.9388	0.9694
inhibition	98	99	0.9899	0.9899	0.8384	0.9697	0.0101	0.9899	0.9697	0.8283
japanese	73	79	0.9241	0.9241	0.6329	0.9211	0.1646	0.8947	0.6329	0.9367
lead	27	29	0.9310	0.9310	0.8276	0.3793	0.9310	0.1724	0.8276	0.8621
man	58	92	0.6304	0.8696	0.6522	0.3187	0.3187		0.6484	0.4176
mole	83	84	0.9881	0.9881	0.4405	0.8916	0.8889	0.9398	1.0000	1.0000
mosaic	57	97	0.5876	0.8247	0.8144	0.5795	0.5979	0.7273	0.8454	0.7216
nutrition	45	89	0.5056	0.5506	0.3708	0.3933	0.5056	0.4719	0.4545	0.4318
pathology	85	99	0.8586	0.8586	0.6061	0.3939	0.8404	0.8182	0.7553	0.8298
pressure	96	96	1.0000	0.9580	0.5208	0.9836	0.1042	0.8172	0.6354	0.8750
radiation	61	98	0.6224	0.8367	0.7449	0.6979	0.7551	0.7917	0.7653	0.7653
reduction	9	11	0.8182	0.8182	0.9091	0.8182	0.9091	0.8182	1.0000	0.8182
repair	52	68	0.7647	0.9559	0.8529	0.8358	0.6618	0.8358	0.8676	0.8824
resistance	3	3	1.0000	1.0000	1.0000	0.3333	0.0000	1.0000	1.0000	1.0000
scale	65	65	1.0000	1.0000	0.7231	0.0615	1.0000	0.0615	0.6875	0.6563
secretion	99	100	0.9900	0.9900	0.4600	0.3535	0.9451	0.9798	0.5814	0.9651
sensitivity	49	51	0.9608	0.9608	0.7255	0.8431	0.0196	0.2745	0.9216	0.7255
sex	80	100	0.8000	0.8400	0.6000	0.5455	0.2700		0.6000	0.5300
single	99	100	0.9900	0.9900	0.8900	0.0400	0.9700	0.9300	0.8900	0.9500
strains	92	93	0.9892	0.9892	0.9570	0.9780	0.6129	1.0000	0.9892	0.9570
support	8	10	0.8000	0.8000	1.0000	0.3000	0.2000	0.9000	1.0000	0.9000
surgery	98	100	0.9800	0.9800	0.1900	0.9394	0.6900	0.8990	0.4300	0.9600
transient	99	100	0.9900	0.9900	0.9100	0.9900	0.9899	0.9600	0.9485	0.9691
transport	93	94	0.9894	1.0000	1.0000	0.9780	0.0106	1.0000	1.0000	0.9787
ultrasound	84	100	0.8400	0.8500	0.7400	0.6667	0.8500	0.7813	0.8100	0.8300
variation	80	100	0.8000	0.9100	0.6900	0.7600	0.8586	0.3500	0.6465	0.8586
weight	29	53	0.5472	0.8491	0.6604	0.4717	0.6444		0.6591	0.6818
white	49	90	0.5444	0.8111	0.5111	0.4831	0.5393	0.6517	0.5730	0.5843

Accuracy all	68.96	81.35	0.8550	0.8830	0.6836	0.6389	0.5826		0.7626	0.7601
Accuracy JDI set	69.47	81.02	0.8669	0.9063	0.6932	0.6526	0.5867	0.7475	0.7794	0.7739

F Max is the number of instances of the sense with the frequency, Total is the number of instances, MFS stands for Maximum Frequency Sense, NB stands for Naïve Bayes, AEC stands for Automatic Extracted Corpus, MRD stands for Machine Readable Dictionary, PPR s stands for Page Rank and MetaMap with Strict model, CombSW stands for weighted linear combination and CombV stands for voting combination.

(3) $$Accuracy\;=\frac{\left|Instances\;\;Correctly\;\;Predicted\right|}{\left|All\;\;Instances\right|}$$

Naïve Bayes, on the NLM WSD set, has the best performance, showing better performance than the MFS baseline. This has already been shown in the literature [14-16]. The MFS indicates that usually one sense of the ambiguous word is highly represented compared to the rest of the senses. These two baselines require special consideration since information like the sense with the highest frequency is not available for all the concepts in the Metathesaurus and there is not training data available for all the ambiguity cases in the Metathesaurus. This means that a production system cannot be developed based on these baselines but are still interesting as reference for WSD performance. Knowledge-based approaches have a lower performance compared to these methods since they do not have access to this information and rely on the information available in the reference knowledge source, which is usually not optimized to perform disambiguation.

In table 3, we find that the JDI method achieves better performance compared to other knowledge-based methods. The MRD approach produces results not as good compared to AEC. The PPR approach presents the lowest performance compared to any of the approaches presented in this study.

We have presented the performance of knowledge-based approaches with different views on the disambiguation problem. These approaches could complement each other. We have combined the methods either by vote or sum of scores by each individual system. The combination obtained by the sum of scores, when no weight is applied, is biased towards the AEC approach. As the JDI method is the best performing one, we have combine the methods providing a higher weight to the JDI method.

Results in tables 3 and 4 indicate that combining approaches results in an improvement of the performance compared to the best performing method. This improvement shows as well that the methods complement each other. Compared to JDI, the combination provides a larger improvement in cases like *lead*, *frequency*, *scale *or *sensitivity*. But performance decreased significantly in cases like *determination *when using a voting combination and in cases like *surgery *and *energy *when applying the linear combination.

The PPR approach has a lower performance compared to other approaches presented in this work. We initially avoided this approach in the combination of methods, but the final result was better than when the system was added.

The JDI method has the best performance with *determination*, *failure*, *fit*, *resistance*, *strains *and *transport*. Some of these terms match some of the best performing methods for the other methods as shown below. The terms with the lowest performance are: *scale*, *lead*, *frequency*, *blood pressure *and *sensitivity*. We find several possible explanations for this behaviour. The JDI method performs JD indexing on the context of the ambiguous words. Sometimes the context might not provide enough evidence to produce a reasonable indexing. Another issue might be related to the indexing of semantic types with JDs.

We have observed that some of the semantic types have as top ranked a set of JDs which do not look as relevant to the category. An example is the semantic type *Temporal Concept *for which the top ranked JDs are: *Gynecology*, *Obstetrics*, *Endocrinology*, *Metabolism *and *Reproductive Medicine*. Another example is *Intellectual Property *for which the top ranked JDs are: *Tropical Medicine*, *Medical Informatics*, *Epidemiology*, *Communicable Diseases *and *Veterinary Medicine*. This might mean that for some semantic types either there are no appropriate JDs to index them or that the terms collected as features from the Metathesaurus do not provide enough evidence for a proper indexing.

The automatically extracted corpus seems to produce better performance than the MRD and PPR approach. There are several possible explanations for this. The MRD relies on the terms presented in the dictionary, in this case the UMLS Metathesaurus. We identify related terms, but in some cases these terms are not representative of the context for a given sense. On the other hand, the automatically extracted corpus seems to rely on the UMLS content to collect documents from MEDLINE which might expand the context terms and, in addition, rely on statistical learning approaches which might produce a better partition of the feature space.

Among the best performing terms with the automatically extracted corpus we find: *transport*, *support*, *resistance*, *depression *and *strains*. These cases are homonyms (not polysemous); so their senses are easy to identify. On the other hand, the terms with the lowest performance are: *growth*, *determination*, *surgery*, *nutrition *and *blood pressure*. In these cases, the differences are blurred and seem to be closer in meaning. An exception is the term *growth*, where the set of terms extracted from the Metathesaurus for the concept linked to the M2 (*Functional Concept*) sense are contained within the terms extracted from the Metathesaurus for the M1 (*Organism Function*) sense. So, the extracted citations might be related for both senses.

The term *blood pressure *in the Metathesaurus could indicate the *blood pressure determination *procedure or the *blood pressure *level of a patient. These senses are difficult to distinguish and the UMLS did not provide enough information to generate a query for each concept denoting *blood pressure *which would allow retrieving distinctive citations to train a classifier to perform WSD.

The term *cold *shows a low performance as well. This is strange since the annotators found that the senses for *cold *were clearly distinct in the documents. Looking at the confusion matrix of the Naïve Bayes classifier, we have found that a large proportion of instances belonging to the sense M1 (*Cold Temperature*) have been classified as M5 (*Cold Sensation*). Looking at the retrieved documents we find as well that one of the assumptions by Yarowsky (one sense per document) does not hold in the *cold *case since multiple meanings of the term cold happen in the documents retrieved for the sense M5. Further refinement of the terms in the UMLS Metathesaurus might retrieve better documents since terms for *cold temperature *did not retrieve some of the documents. Since the term *cold *might have several meanings in the same document, disambiguation approaches looking at a narrower word context might improve the results. The automatically generated queries are not specific enough in some cases, so they retrieve false positives for a given sense. The results are in tune with general English results, where the performance is lower than using manually generated training data. We identify similar cases where senses are not so clearly distinct. On the other hand, these cases are more difficult to spot from text compared to similar tasks in the biomedical domain where acronyms are the ambiguous terms to disambiguate [21] and the long form is used to identify the correct term. Specific needs for WSD could be studied with these techniques. Identifying further heuristics for a more general disambiguation approach is welcome.

Among the terms for which the MRD approach has the best performance we find *depression*, *determination*, *transport*, *strains *and *transient*. Some of these terms match the ones from the automatically extracted corpus. In the case of *transport*, in the biological sense terms like *process *or *metabolism *are within the most relevant terms and in the patient sense we find *patient *and *delivery*. The context defined by the concept vectors allows properly differentiating the sense in text.

On the other hand there are some cases in which the MRD approach cannot disambiguate properly. Among these cases we find: *single*, *scale*, *nutrition*, *adjustment *and *extraction*. Despite the fact that some of the terms might be confusing in context (e.g. *man*), in these cases, the concept profiles might not be representative of the ambiguous term senses. So, the terms with higher *tf *× *idf *are not representative of the context of the ambiguous words.

Considering the term *scale*, the sense M2 (*Intellectual Scale*) has the highest frequency in the benchmark but the MRD approach seems to prefer the sense M1 (*Integumentary Scale*). Looking at the concept profiles in table 5, we find that the terms in M2 do not really seem to contain terms which could co-occur with *scale *in the M2 context. In addition, the vector for M1 is very short, containing two dimensions (*integumentary*, *scale*), so matching the term *scale *biases the sense prediction to M1.

**Table 5:** Scale top tf × idf terms for the senses M1, M2, M3

M1		M2		M3	
**Term**	**tf × idf**	**Term**	**tf × idf**	**Term**	**tf × idf**

scale	19.06	scale	68.75	scale	55.30
integumentari	8.39	interv	25.17	weight	46.06
		seri	24.74	measur	41.91
		loinc	22.52	compon	33.80
		sequenc	21.38	devic	31.98

In the case of *nutrition*, which also has low performance in the noisy-corpus approach, we find that the vectors have similar terms with high *tf *× *idf *(c.f. table 6). In the WSD results, we find that the correct senses are split among M1 (*Organism Attribute*) and M3 (*Feeding and dietary regimes*), and no ambiguous term is assigned to M2 (*Science of Nutrition*). The MRD approach classifies M3 cases as M2 or M1, and some M1 cases are assigned to M2; but no annotation is made to M3.

**Table 6:** Nutrition top tf × idf terms for the senses M1, M2, M3

M1		M2		M3	
**Term**	**tf × idf**	**Term**	**tf × idf**	**Term**	**tf × idf**

nutrit	1519.81	nutrit	1318.84	nutrit	158.13
physiolog	548.57	scienc	453.13	Scienc	81.95
avail	205.97	health	433.43	Statu	35.07
Statu	182.38	physiolog	351.14	Regim	13.48
phenomena	131.35	food	311.01	Outcom	10.17

The page rank approach (PPR) has inferior performance compared to the other methods. One possible reason is the assumption that concepts having more related concepts might also be more relevant does not hold for the UMLS. The terms with the best performance have a large number of relations linked to the right sense; this means that there is a large number of concepts linked to that sense. These best performing terms are: *frequency*, *fit*, *transient*, *single *and *scale*. Most of these well performing terms seem to be performing well with the other methods as shown above. Looking at the Metathesaurus we find that in most of the cases these terms are connected to a larger number of concepts compared to other candidate concepts. The terms with the worst performance are: *determination*, *resistance*, *inhibition*, *transport *and *sensitivity*. Terms like *inhibition*, *sensitivity *and *determination *present a significantly lower number of relations associated to the concept with the maximum frequency in the data set. The Metathesaurus provides many types of relations and many of them might not be relevant to WSD. We have tried to remove some of these relations because they either add noise or duplicate information from other relation types. Among these relations we can find *QB *(can be qualified by) and *AQ *(Allowed Qualifier). In addition, taxonomic relations are expressed with different relation types which might be duplicated in the relation file. For instance, *CHD *(has child) and *PAR *(has parent) express parent child relations *SIB *(has sibling) which might be implied by other taxonomic relations. We have tried to remove some of these relations from the relation file but the result in disambiguation did not change significantly. This implies that the number of relations in the Metathesaurus does not directly imply relevance of the sense to perform WSD. The term *resistance *is special compared to the other terms because the concepts in the Metathesaurus are related and this might have caused the preference of a concept over the other.

## Conclusions

We have compared several methods for knowledge-based sense disambiguation in the biomedical domain. We find that the JDI method has the best performance for a single method. Indexing of semantic types and ambiguous words' contexts to Journal Descriptors seem to perform well using the NLM WSD data set. Further research on indexing of some semantic types might improve the performance of the method.

In addition, MRD and PPR seem to have a lower performance than the AEC approach. The AEC approach has collected contextual features which perform better compare to the use of features extracted from the UMLS by the MRD and PPR approaches. It is not the purpose of the UMLS to perform WSD and we can foresee some research to produce a UMLS version tuned for WSD.

The graph-based method (PPR) has shown to perform less well than other methods. This means that statistics estimated from the graph structure of the Metathesaurus might not reliably imply relevance. The combination of the predictions of the methods compared in this work performs better than any individual method. This increase in performance shows that the methods have complementary views of the data.

Automatic extraction of a corpus from MEDLINE seems to provide good results but still has some drawbacks. Filtering of documents to improve the quality of the automatically extracted corpus could improve the performance of the statistical learning algorithms on the automatically extracted corpus. Corpus statistics might help to complement the UMLS and improve WSD methods or related text mining tasks. For example, corpus statistics might help to optimize the UMLS Metathesaurus to improve document retrieval from MEDLINE. Several ideas have already been proposed to clean up an existing thesaurus [8,9,34] and to add further relevant content [35].

Knowledge-based approaches achieve good performance, even though below standard WSD baselines. We have presented several approaches and analyzed their performance and drawbacks. Finally, we have proposed several directions for further research which might improve performance, and some of these could be used to improve the UMLS for WSD.

## Authors' contributions

AJ designed and carried out the experiments, participated in the development of the methods and drafted the manuscript. AA designed the experiments and reviewed the manuscript. AJ and AA read, commented, and approved the final version of the manuscript.
